# Therapeutic potential of melatonin in the intervertebral disc degeneration through inhibiting the ferroptosis of nucleus pulpous cells

**DOI:** 10.1111/jcmm.17818

**Published:** 2023-06-16

**Authors:** Xinyu Dou, Yunlong Ma, Qipeng Luo, Chunyu Song, Meijuan Liu, Xiao Liu, Donglin Jia, Shuiqing Li, Xiaoguang Liu

**Affiliations:** ^1^ Department of Orthopaedics Peking University Third Hospital Beijing China; ^2^ Beijing Key Laboratory of Spinal Disease Research Beijing China; ^3^ Engineering Research Center of Bone and Joint Precision Medicine Ministry of Education Beijing China; ^4^ Pain Medical Center Peking University Third Hospital Beijing China; ^5^ Department of Anesthesiology Peking University Third Hospital Beijing China; ^6^ Department of Endocrinology, Genetics and Metabolism Beijing Children's Hospital Beijing China

**Keywords:** ferroptosis, inflammation, intervertebral disc degeneration, macrophage, melatonin, nucleus pulposus cell

## Abstract

Ferroptosis, a novel type of cell death mediated by the iron‐dependent lipid peroxidation, contributes to the pathogenesis of the intervertebral disc degeneration (IDD). Increasing evidence demonstrated that melatonin (MLT) displayed the therapeutic potential to prevent the development of IDD. Current mechanistic study aims to explore whether the downregulation of ferroptosis contributes to the therapeutic capability of MLT in IDD. Current studies demonstrated that conditioned medium (CM) from the lipopolysaccharide (LPS)‐stimulated macrophages caused a series of changes about IDD, including increased intracellular oxidative stress (increased reactive oxygen species and malondialdehyde levels, but decreased glutathione levels), upregulated expression of inflammation‐associated factors (IL‐1β, COX‐2 and iNOS), increased expression of key matrix catabolic molecules (MMP‐13, ADAMTS4 and ADAMTS5), reduced the expression of major matrix anabolic molecules (COL2A1 and ACAN), and increased ferroptosis (downregulated GPX4 and SLC7A11 levels, but upregulated ACSL4 and LPCAT3 levels) in nucleus pulposus (NP) cells. MLT could alleviate CM‐induced NP cell injury in a dose‐dependent manner. Moreover, the data substantiated that intercellular iron overload was involved in CM‐induced ferroptosis in NP cells, and MLT treatment alleviated intercellular iron overload and protected NP cells against ferroptosis, and those protective effects of MLT in NP cells further attenuated with erastin and enhanced with ferrostatin‐1(Fer‐1). This study demonstrated that CM from the LPS‐stimulated RAW264.7 macrophages promoted the NP cell injury. MLT alleviated the CM‐induced NP cell injury partly through inhibiting ferroptosis. The findings support the role of ferroptosis in the pathogenesis of IDD, and suggest that MLT may serve as a potential therapeutic approach for clinical treatment of IDD.

## 1 INTRODUCTION

Intervertebral disc degeneration (IDD) is a leading cause of low back pain, which affects almost 80% of the global population at least one time during their life.^1^ Although the pathogenesis has not been clearly defined, it is accepted that the progression of IDD is initiated and accelerated by the depletion of nucleus pulposus (NP) cells and degradation of extracellular matrix (ECM). Therefore, it will be of great clinical significance to elucidate the pathogenetic mechanisms underlying IDD via investigating NP cells. NP cell, a main type of cell resident in the NP of the intervertebral disc (IVD), is critically important for maintaining the physiological function of the IVD. NP cells are responsible for the synthesis and decomposition of the ECM, which, in turn, maintains the normal structural and functional properties of the IVD. A previous study demonstrated that the anabolism/catabolism molecules of ECM like collagen type II α1 (COL2A1), aggrecan (ACAN), matrix metalloproteinase‐13 (MMP‐13), thrombospondin type 1 motif 4 (ADAMTS4) and thrombospondin type 1 motif 5 (ADAMTS5) are changed in the degenerated IVD tissue.^2^ Additionally, the degree of oxidative stress in the degenerative IVD is aggravated and the levels of oxidative stress‐associated markers, including reactive oxygen species (ROS), malondialdehyde (MDA) and reduced glutathione (GSH), are also altered in the degenerative IVD.^3^ While the exact aetiology of IDD remains undetermined, the loss of NP cells and the subsequent degradation of the ECM are considered as the major pathological characteristics during the development of IDD.^4^ Oxidative stress not only triggers inflammation and matrix degradation, but also promotes the decrease in the number of viable and functional cells in the micro‐environment of the IVD. Moreover, increasing evidence has confirmed that the loss of NP cells due to the apoptosis, autophagic and necrosis plays a critical role in the pathological development of IDD.^5^, ^6^ Ex vitro studies have demonstrated that the biologic interactions between NP cells and activated macrophages formed a positive feedback loop, leading to severe inflammation, which subsequently induced the synthetic and degraded imbalance of ECM and finally exacerbated the IDD development.^7^ Therefore, amelioration of NP cell injury via targeting the inflammatory macrophages and the modulation of cell death of NP cells, might be a potential and attractive therapeutic strategy for the treatment of IDD in clinic.

Ferroptosis, discovered by Stockwell et al. in 2012, is an emerging new type of programmed cell death, which is morphologically, biochemically and genetically distinct from apoptosis, autophagy and necrosis.^8^ Mechanistically, ferroptosis is caused by the elevation of intracellular iron levels and the accumulation of lipid ROS.^8^ Studies have shown that ferroptosis was involved in the pathological process of various diseases, including carcinogenesis, stroke, intracerebral haemorrhage, traumatic brain injury, ischemia–reperfusion injury, kidney injury and degenerative diseases such as Parkinson's, Huntington's, and Alzheimer's diseases.^9^ More importantly, clear evidence has consistently demonstrated that ferroptosis contributed to the pathogenesis of IDD.^10^, ^11^ In clinic trials, the proteins markers of ferroptosis, such as the levels of glutathione peroxidase 4 (GPX4) expression and solute carrier family 7 member 11 (SLC7A11) were significantly decreased, while acyl‐CoA‐synthetase long‐chain family number 4 (ACSL4) was increased in the degenerated IVD tissue compared with the normal tissue.^10^, ^11^ Animal studies showed that the degree of disc degeneration was attenuated in the iron chelator deferoxamine (DFO) group and ferroptosis inhibitors ferrostatin‐1 (Fer‐1) relative to the saline group.^11^ Therefore, key factors in ferroptosis‐related pathways like SLC7A11, GPX4, ACSL4 and lysophosphatidylcholine acyltransferase 3 (LPCAT3) played an important role in multiple diseases including tissue injury, inflammation and IDD.^12^

Melatonin (*N*‐acetyl‐5‐methoxytryptamine, MLT), is an indoleamine hormone, which displays a variety of biological properties, including anti‐oxidant,^13^, ^14^ anti‐inflammatory,^15^, ^16^ anti‐apoptosis,^17^, ^18^ anti‐cancer,^19^ anti‐diabetic^20^ and immunomodulation.^21^, ^22^ Recently, clinical and animal studies suggested the role of MLT in the IDD.^3^, ^23^, ^24^, ^25^, ^26^, ^27^, ^28^, ^29^ In patients with IDD, the level of plasma MLT concentration was significantly lower in comparison with that of healthy control group, which was correlated with the disease duration, severity and the levels of inflammatory cytokines.^24^ Animal studies revealed the importance of MLT in IDD, that exogenous MLT administration activated the recovery process in the degenerated IVD tissue in rats^3^ and rabbits^29^; whereas surgical pinealectomy significantly reduced serum MLT levels in chicken, resulted in the accelerated deterioration of IDD.^30^ Additionally, MLT was an effective inhibitor of ferroptosis and its anti‐ferroptosis provided a potential therapeutic target for treating traumatic brain injury and acute sleep deprivation‐induced cognitive impairment.^31^, ^32^ However, whether MLT ameliorates NP cell injury through inhibiting ferroptosis remains unclear. To determine the clinical translational potential of melatonin for the treatment of IDD, the conditioned medium (CM) from the lipopolysaccharide (LPS)‐stimulated RAW264.7 macrophages was utilized to induce the injury of primary mouse NP cells. Current mechanistic studies demonstrated that MLT could attenuate NP cell injury through the inhibition of the ferroptosis in NP cells, providing the evidence to optimize the clinical management of IDD with MLT.

## 2 MATERIALS AND METHODS

### 2.1 Isolation and primary culture of mouse NP cells

IVDs were harvested from the lumbar spines of C57BL/6 mice immediately after they were euthanized according to the IACUC animal protocol approved by Peking University Third Hospital Ethics Committee (No. A2019021). The NP tissues were carefully separated from the annulus fibrosus under the microscope and cut into small fragments. Fragments were digested with 0.25% Type II collagenase (Sigma) for 1 h, followed by trypsin–EDTA solution (0.2% trypsin, 1 mM EDTA, Gibco) for 5 min, respectively. Then the isolated NP cells were cultured at 37°C under 5% CO2 conditions in Dulbecco's modified Eagle medium (DMEM, HyClone, Thermo Scientific, Logan, UT, USA) containing 20% foetal bovine serum (FBS, Gibco, Grand Island, NY, USA). When NP cells reached 80%–90% confluency, they were passaged with trypsin–EDTA solution (0.25% trypsin, 1 mM EDTA, Gibco). The third passage (P3) NP cells were applied for the subsequent experiments.

### 2.2 Preparation of CM

To activate the polarisation of macrophages, macrophages were stimulated by lipopolysaccharide (LPS).^33^ Briefly, RAW 264.7 macrophage cells (the National Experimental Cell Resource Sharing platform, Beijing, China) CM was prepared as follows: RAW 264.7 macrophage cells were seeded at 4 × 106 in 10‐cm tissue culture‐treated dishes. They were cultured with DMEM completed medium overnight before being treated with 1 μg/mL lipopolysaccharide (LPS, Escherichia coli serotype 0111: B4, Sigma‐Aldrich) for 24 h. Cell supernatant was discarded and RAW 264.7 macrophage cells were washed three times with phosphate buffer saline (PBS, HyClone, Thermo Scientific). After removing the supernatant, RAW 264.7 macrophage cells were cultured again in DMEM completed medium for another 24 h. The cell culture medium was then collected and centrifuged to remove cell debris. The remaining supernatant was designated as CM and utilized for subsequent experiments.

### 2.3 MTT assay

The cytotoxicity of MLT on NP cells was evaluated by the MTT assay (Sigma‐Aldrich). MLT was purchased from Rhawn and dissolved in dimethyl sulphoxide (DMSO). NP cells were seeded in a flat‐bottom 96‐well plate at an initial density of 6 × 103 cells/well and incubated at 37°C, and 5% CO_2_ conditions. After 24 h, cells were treated with 0.01, 0.1, and 1 μM concentration of melatonin respectively. Cells treated with only 0.1% DMSO served as a negative control. Such treated NP cells were incubated in a humidified environment with 5% CO_2_ at 37°C for 24 h. The MTT reagent (10 μL) was then added to each well and incubated at 37°C for 4 h. Then the medium in each well was removed after centrifuge and followed by adding 200 μL DMSO solution before being gently agitated for 10 min at room temperature. The absorbance at 490 nm was read using a microplate reader (Thermo Scientific, Vario Skan Flash).

### 2.4 ROS, MDA and GSH assays

Intracellular ROS levels of NP cells were detected using a ROS detection assay kit (Beyotime) according to the manufacturer's instructions. NP cells were seeded at a density of 2 × 105 cells/well in a six‐well cell culture plate and were treated with the CM from RAW 264.7 macrophage cells for 24 h at 37°C under 5% CO_2_ conditions. Then cells were treated with 0.1 μM of MLT, 5 μM of ferroptosis inducer erastin (Selleckchem), or 1 μM of ferroptosis inhibitor ferrostatin‐1 (Fer‐1, Selleckchem) respectively for 24 h. Treatment with DMEM completed medium served as a negative control. Next, cells were incubated with 2′,7‐dichlorofluorescein diacetate (DCFH‐DA, 10 μM) for 20 min at 37°C. Finally, the cells were washed three times with a serum‐free medium, after which DCF fluorescence intensity was measured by a biological inverted microscope (Olympus Co.) and a Becton–Dickinson FACSCalibur (BD Biosciences).

The content of MDA was analysed using a commercial kit (Beyotime) according to the manufacturer's instructions. The harvested cells were lysed for 60 min and rotated at 12,000 × ***g*** for 10 min at 4°C and the supernatant was collected. The protein concentration of the supernatant was detected using a bicinchoninic acid (BCA) kit (Beyotime). Next, 100 μL supernatant was placed into a centrifuge tube, into which 200 μL MDA testing solution was added. After being boiled for 15 min, the mixture was centrifuged at 1000 × ***g*** for 10 min. Afterwards, 200 μL of the supernatant was loaded into a 96‐well plate and absorbance was read at 532 nm with a microplate reader (Rayto).

GSH was measured by using the GSH detection kit (Beyotime) according to the manufacturer's protocols. The cells were washed twice with PBS, harvested, mixed with 30 μL protein removal reagent M, and then performed the freeze–thaw cycles twice with the liquid nitrogen and a water bath at 37°C. After centrifugation at 10,000 × ***g*** for 10 min at 4°C, the supernatant was collected and used for GSH detection. The absorbance of each well at the wavelength of 412 nm was determined using a microplate reader (Rayto).

### 2.5 Intracellular iron determination

The intracellular iron content was determined using the colorimetric Iron Assay kit (Nanjing Jiancheng Bioengineering Institute, Nanjing, China) following the manufacturer's instructions. Briefly, 0.5 mL of cell lysate was mixed with 1.5 mL of the determination buffer and was heated for 5 min in a boiling water bath. Then the mixture was cooled to room temperature with flowing water before being centrifuged at 3500× rpm for 10 min. The OD of the final mixtures was measured at a wavelength of 570 nm using a microplate reader (Rayto).

### 2.6 Real‐time quantitative polymerase chain reaction (qPCR)

Total RNA was extracted from NP cells using Trizol reagent (TransGen Biotech) according to the manufacturer's instructions. Total RNA (1 μg) was reverse‐transcribed into cDNA by using the Quantscript RT Kit (Beyotime) according to the manufacturer's protocol. The cDNA was then used as the template for the qPCR with TransStart® Top Green qPCR SuperMix kit (TransGen Biotech) on the Eppendorf Realplex4 instrument (Eppendorf). The reaction conditions were set as follows: 95°C for 3 min, followed by 40 cycles at 95°C for 30 s and at 55°C for 20 s, and extension for 20 s at 72°C. All primer sequences are listed in the Table 1. The 2^−ΔΔCt^ method was used to calculate the relative RNA expression levels of target genes.^34^

**TABLE 1:** The list of primers was utilized for qPCR.

Gene	Forward primer (5′‐ to ‐3′)	Reverse primer (5′‐ to ‐3′)
*IL‐1β*	GGACCCATATGAGCTGAAAG	TCCACTTTGCTCTTGACTTC
*COX2*	CATAAGCGAGGACCTGGGTT	TGGCATACATCATCAGACCA
*iNOS*	CTGGACAAGCTGCATGTGAC	TGGGTCCTCTGGTCAAACTC
*ACAN*	GAACTTGGCCATGGTCCTTC	TGTTTACCTCTGTGCTTGGG
*COL2A1*	TGAAGGGTGAGAGTGGTTCC	ACGAGAACCTTGAGCACCTT
*MMP‐13*	TCTATGATGGCACTGCTGAC	GTTGTAGCCTTTGGAACTG
*ADAMTS4*	CATCACTGACTTCCTGGACA	CGAAGGTCAGTTGGCATTG
*ADAMTS5*	GGCAGACGTTGGGACCATAT	TCTGTGATGGTGGCTGACGT
*GPX4*	CACGAATTCTCAGCCAAGGA	AACCACACTCAGCATATCGG
*SLC7A11*	CTACTGCTGTGATATCCCT	GCTGTATAACTCCAGGGACT
*ACSL4*	AACCCCTTCAGACATGGCCA	TACAATCACCCTTGCTTCCC
*LPCAT3*	CCCACATCACAGACGACTAT	CCGACAGAATGCACACTCCT

### 2.7 Western blot

NP cells were lysed with RIPA lysis buffer (Beyotime), and the protein concentration in cell lysates was measured by BCA standard method (Beyotime). 100 μg protein samples were separated by 10% SDS‐PAGE and transferred to a polyvinylidene difluoride (PVDF) membrane (Millipore). The PVDF membranes were blocked with 3% bull serum albumin (BSA) for 2 h at room temperature after being washed with tris buffered saline tween (TBST, 0.05% Tween‐20) three times. Then, the PVDF membranes were incubated with the primary antibodies at 4°C overnight. Subsequently, the prepared membranes were incubated with corresponding secondary antibodies at room temperature for 2 h after being washed with TBST three times. The blots were detected using an enhanced chemiluminescence kit (Beyotime) and the signal intensity was quantified with Image J software. The following primary polyclonal antibodies were used: rabbit anti‐mouse IL‐6 (Cat. No. PAA079Mu01), rabbit anti‐mouse IL‐1β (Cat. No. PAA563Mu01), rabbit anti‐mouse TNF‐α (Cat. No. PAA133Mu01), rabbit anti‐mouse iNOS (Cat. No. PAA837Mu01), rabbit anti‐mouse COX2 (Cat. No. PAA699Mu01), rabbit anti‐mouse ACAN (Cat. No. PAB908Mu01), rabbit anti‐mouse COL2A1 (Cat. No. PAD194Mu01), rabbit anti‐mouse MMP‐13 (Cat. No. PAA099Mu01), rabbit anti‐mouse GPX4 (Cat. No. PAC994Mu01), rabbit anti‐mouse LPCAT3 (Cat. No. PAG531Mu01) that from the Cloud Clone Corporation, Wuhan, China; rabbit anti‐mouse ADAMTS‐4 (Cat. No. ab185722), rabbit anti‐mouse ADAMTS‐5 (Cat. No. ab41037) that from the Abcam, Cambridge, England; rabbit anti‐mouse anti‐ACSL4 (Cat. No. ab2844946) that from the Affbiotech, Jiangsu, China; and rabbit anti‐mouse SLC7A11 (Cat. No. BM5318) that from the Boster Biological Technology Co., Ltd, Wuhan, China.

### 2.8 Transmission electron microscopy (TEM) of mouse NP cells

The ultrastructure of mice NP cells after the treatment with different approaches (Negative control/CM‐treated/CM + MLT‐treated/CM + MLT + Erastin‐treated/CM + MLT + Fer‐1‐treated) was examined by TEM. Briefly, cells were collected after washing twice with PBS and fixed with 2.5% glutaraldehyde (Beyotime) overnight at 4°C, and followed by washing with PBS and postfixed in 1% (wt/vol) osmium tetroxide, dehydrated by ethanol gradient, and finally embedded in resin. Ultrathin sections (70 nm) were double‐stained with uranyl acetate and lead citrate. Cells were observed and photographed with TEM (JEOL JEM‐1010, JEOL).

### 2.9 Statistical analysis

Data were presented as mean ± SD. Comparison of continuous data between the two groups was performed by independent Student's *t*‐test, which was analysed by using IBM SPSS software version 20 (IBM Corp.). Differences at *p* <0.05 were considered statistically significant.

## 3 RESULTS

### 3.1 MLT displays no cytotoxicity on NP cells

**FIGURE 1:**
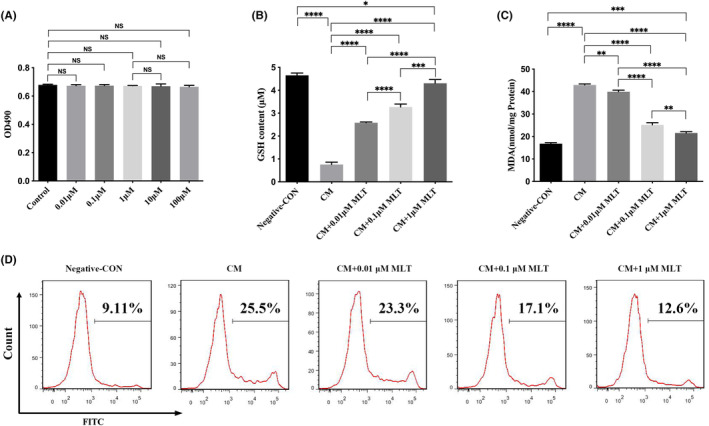
Protective effects of MLT against the oxidative stress in nucleus pulposus (NP) cells. NP cells were treated with conditioned medium (CM) in the presence or absence of MLT for 24 h, and followed by different testing. (A) Melatonin displayed no cytotoxicity effects in NP cells, at different concentrations of MLT ranged from 0.01 up to 100 μM (*n* = 3). (B–D) Reduction of oxidative stress by MLT in NP cells. (B) MLT increased the level of glutathione (GSH). (C) MLT decreased the level of malondialdehyde (MDA). (D) The reactive oxygen species (ROS) levels were declined after treatment with different concentrations of MLT. The levels of GSH and MDA in NP cells were determined by ELISA (*n* = 3). The intracellular ROS levels in NP cells were measured by flow cytometry assay. Data are expressed as mean ± standard deviation. The two groups among the five groups are compared by using an independent *t*‐test. *p < 0.05, **p < 0.01, ***p < 0.001, ****p < 0.0001.

To determine whether melatonin display the effects of cytotoxicity, NP cells were treated with 0.01, 0.1, 1, 10 and 100 μM MLT respectively for 24 h. MTT assay failed to show the marked cytotoxic effects of different dosages of MLT on NP cells relative to the control group, which ranged from 0.01 to 100 μM (*p* > 0.05) (Figure 1A **)**. However, there is a downward trend in the specific quantity value in groups which ranged from 0.01 to 100 μM, especially in the 10 μM MLT group and 100 μM MLT group. Therefore, MLT at the dosages of 0.01, 0.1 and 1 μM were utilized in the following experiments.

### 3.2 Reduction of oxidative stress by MLT in NP cells

To explore whether MLT exhibit the protective effects on NP cells against the oxidative stress, NP cells were treated with CM from the LPS‐stimulated RAW 264.7 macrophages in the presence or absence of MLT treatment. As shown in Figure 1, the NP cells cultured with CM had a significant decrease in levels of GSH (Figure 1B), while MDA (Figure 1C) and ROS (Figure 1D) levels increased relative to that of untreated cells. It suggested that CM treatment led to oxidative stress in NP cells. To further determine the antioxidant effect of MLT, NP cells were treated with various concentrations of MLT (0.01, 0.1 and 1 μM) in the presence or absence of CM for 24 h. The data demonstrated that treatment with MLT markedly increased the level of antioxidant enzyme GSH (Figure 1B), decreased content of MDA (Figure 1C) and decreased intracellular ROS levels (Figure 1D) in NP cells, in a dose‐dependent manner, relative to those of the MLT‐untreated CM control group. Therefore, these results indicated that MLT treatment significantly protected against the CM‐induced oxidative stress in NP cells.

### 3.3 Protect NP cells against inflammation by the treatment with MLT

**FIGURE 2:**
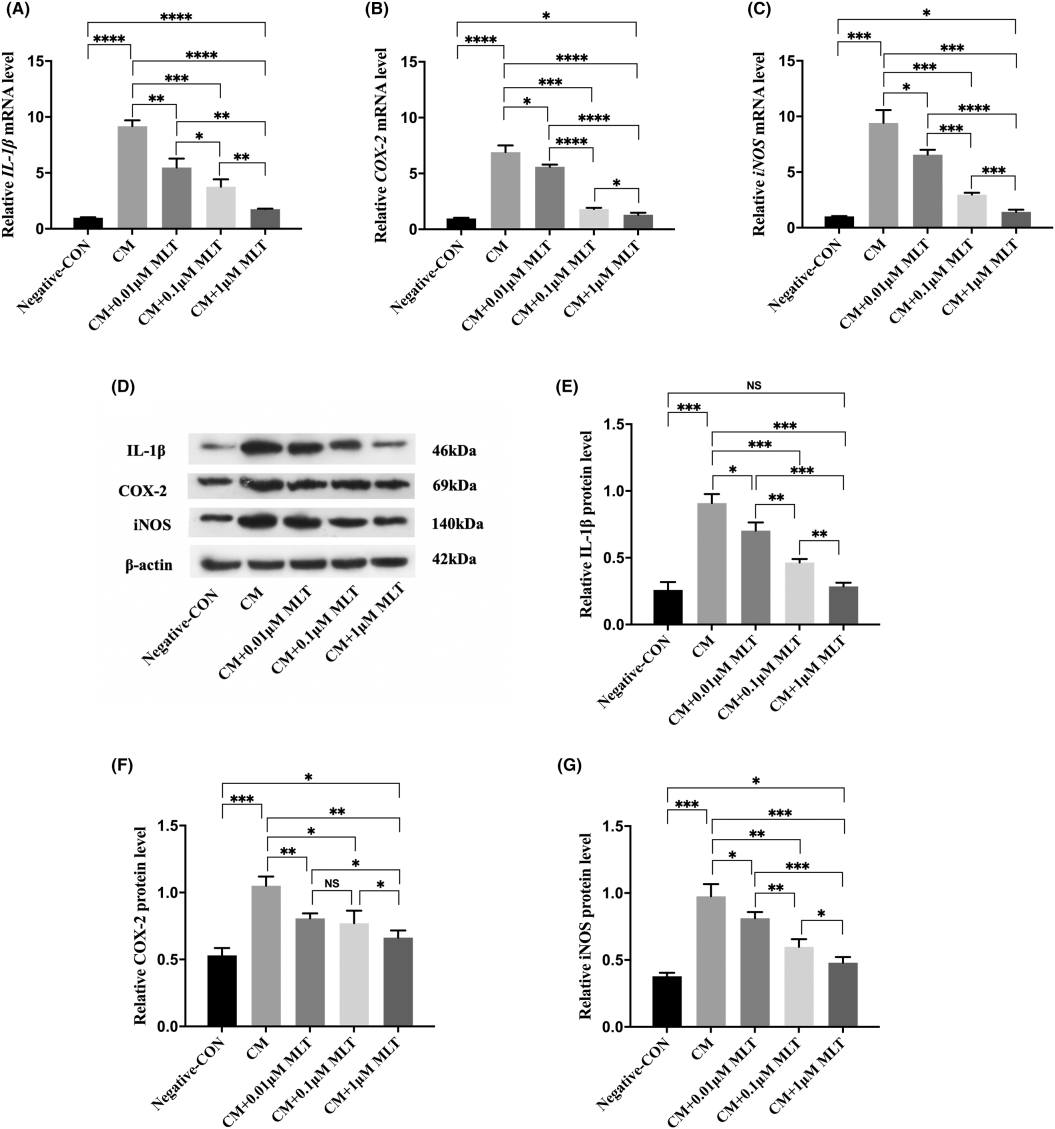
Protect nucleus pulposus (NP) cells against the conditioned medium (CM)‐induced inflammation by the treatment with melatonin (MLT). NP cells were treated with CM in the presence or absence of MLT for 24 h, and followed by different analysis. (A–C) The relative mRNA levels of inflammatory cytokines in NP cells were measured by qPCR analyses (*n* = 3). (A) The levels of IL‐1 β mRNA were decreased in the presence of MLT treatment at different dosages. (B) The levels of COX‐2 mRNA were reduced in the presence of MLT treatment at different dosages. (C) The levels of iNOS mRNA were declined in the presence of MLT treatment at different dosages. (D–G) The relative protein levels of inflammatory cytokines in NP cells were measured by Western blot analyses (*n* = 3). (D) Western blotting showed the downregulation of inflammation‐associated proteins. (E) Quantitative analysis showed the reduced levels of IL‐1β protein after the treatment with MLT at different dosages. (F) Quantitative analysis showed the reduced levels of COX‐2 protein after the treatment with MLT at different dosages. (G) Quantitative analysis showed the reduced levels of iNOS protein after the treatment with MLT at different dosages. Data are expressed as mean ± standard deviation. The two groups among the five groups are compared by using an independent *t*‐test. *p < 0.05, **p < 0.01, ***p < 0.001, ****p < 0.0001.

To further explore the anti‐inflammatory effects of MLT, the other inflammation‐associate factors were examined in the MLT‐treated NP cells by using qPCR at gene levels and Western blot at protein levels. The qPCR analysis showed that compared with negative control NP cells, mRNA levels of pro‐inflammation‐related genes, including IL‐1β, COX‐2 and iNOS were all remarkably upregulated in the CM‐treated NP cells (Figure 2A–C). These results were consistent with those obtained from Western blot (Figure 2D–G), indicating that CM from LPS‐stimulated macrophages evoked the inflammatory reactions in NP cells. In contrast, the mRNA levels of those inflammation‐associated genes in CM + MLT‐treated NP cells were all markedly downregulated relative to those in the MLT‐untreated CM group and this inhibitory effect was in a dose‐dependent manner (Figure 2A–C). Consistent with qPCR results, Western blot further demonstrated that protein levels of these pro‐inflammation cytokines in CM + MLT‐treated NP cells were also significantly declined in comparison with those in the MLT‐untreated CM group (Figure 2D–G). Thus, these data confirmed that treatment with MLT could overcome the inflammation evoked by the CM treatment in NP cells.

### 3.4 Normalize the ECM production of NP cells by the treatment with MLT

**FIGURE 3:**
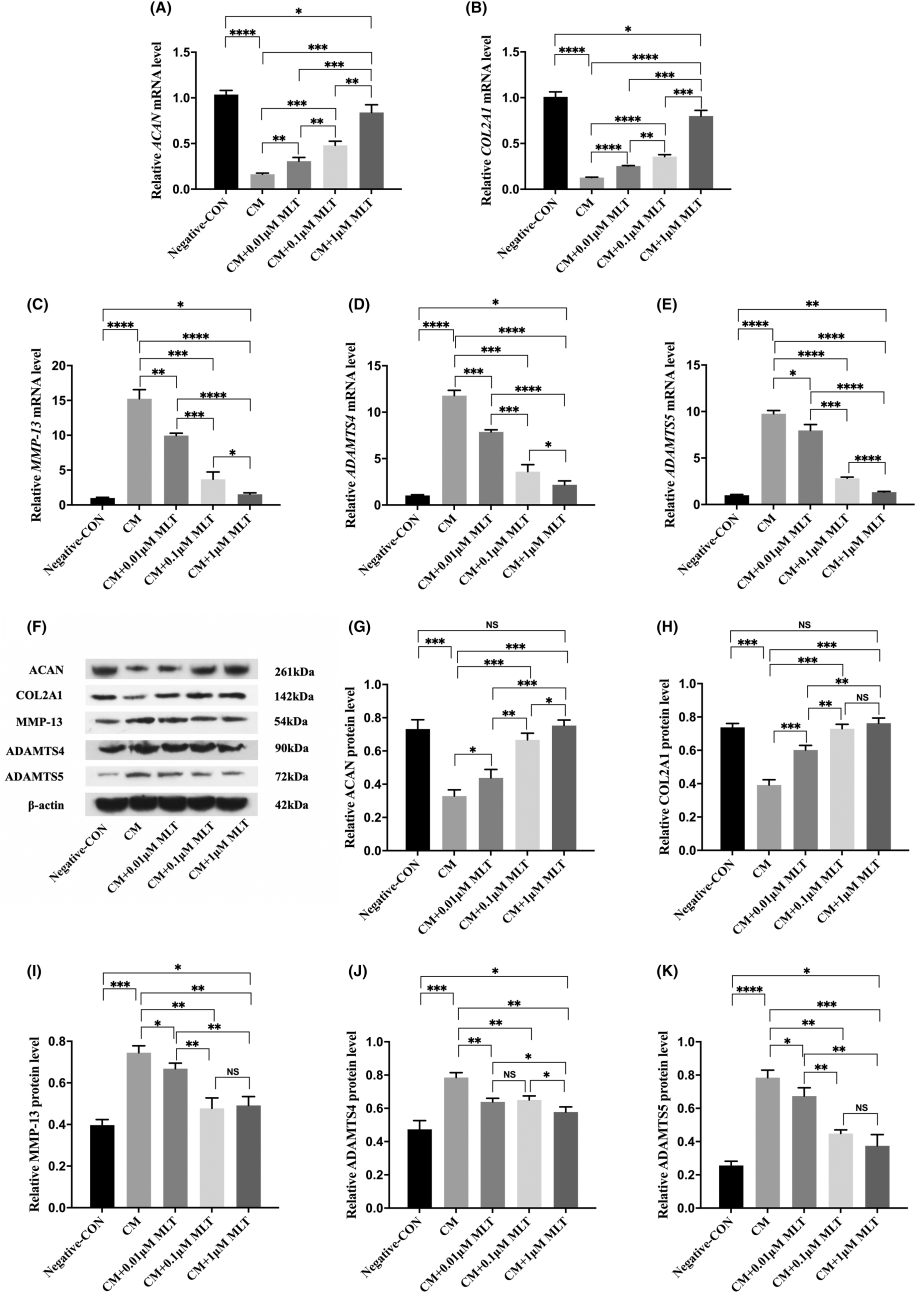
Normalize the extracellular matrix (ECM) production of nucleus pulposus (NP) cells by the treatment with melatonin (MLT). NP cells were treated with conditioned medium (CM) in the presence or absence of MLT for 24 h, and followed by different examinations. (A–E) The relative mRNA levels of matrix anabolic and degrading enzymes in NP cells were measured by qPCR analyses (*n* = 3). (A) The levels of ACNA mRNA were increased in the presence of MLT treatment at different dosages. (B) The levels of COL2A1 mRNA were upregulated in the presence of MLT treatment at different dosages. (C) The levels of MMP13 mRNA were decreased in the presence of MLT treatment at different dosages. (D) The levels of ADAMTS4 mRNA were downregulated in the presence of MLT treatment at different dosages. (E) The levels of ADAMTS5 mRNA were declined in the presence of MLT treatment at different dosages. (F–K) The relative protein levels of matrix anabolic and degrading enzymes in NP cells were measured by Western blot analyses (*n* = 3). (F) Western blotting showed the different modifications of ECM protein productions in NP cells. (G) Western blotting showed the upregulation of ACNA proteins. (H) Western blotting showed the upregulation of COL2A1 proteins. (I) Western blotting showed the reduction of MMP13 proteins. (J) Western blotting showed the downregulation of ADAMTS4 proteins. (K) Western blotting showed the reduced level of ADAMTS5 proteins. Data are expressed as mean ± standard deviation. The two groups among the five groups are compared by using an independent *t*‐test. *p < 0.05, **p < 0.01, ***p < 0.001, ****p < 0.0001.

To evaluate whether the ECM production of NP cells was affected by the CM treatment, the modifications of ECM production of NP cells were analysed by qPCR and Western blot, which include the synthetic‐associated molecules (COL2A1 and ACAN) and ECM degradation‐associated molecules (MMP‐13, ADAMTS4 and ADAMTS5). Comparing with negative control cells, both qPCR and Western blot substantiated that ECM anabolic markers, including COL2A1 and ACAN were significantly decreased, whereas ECM degrading markers, including MMP‐13, ADAMTS4 and ADAMTS5 were significantly increased in the CM‐treated NP cells (Figure 3A–K). However, both qPCR and Western blot revealed that matrix anabolic enzymes, including COL2A1 and ACAN, were markedly upregulated, while matrix degrading enzymes, including MMP‐13, ADAMTS4 and ADAMTS5 were all downregulated in the CM + MLT‐treated NP cells when compared with those in the CM‐treated alone cells (Figure 3A–K). These data indicated MLT could restore the ECM production in NP cells.

### 3.5 Involvement of ferroptosis in CM‐induced NP cell injury

**FIGURE 4:**
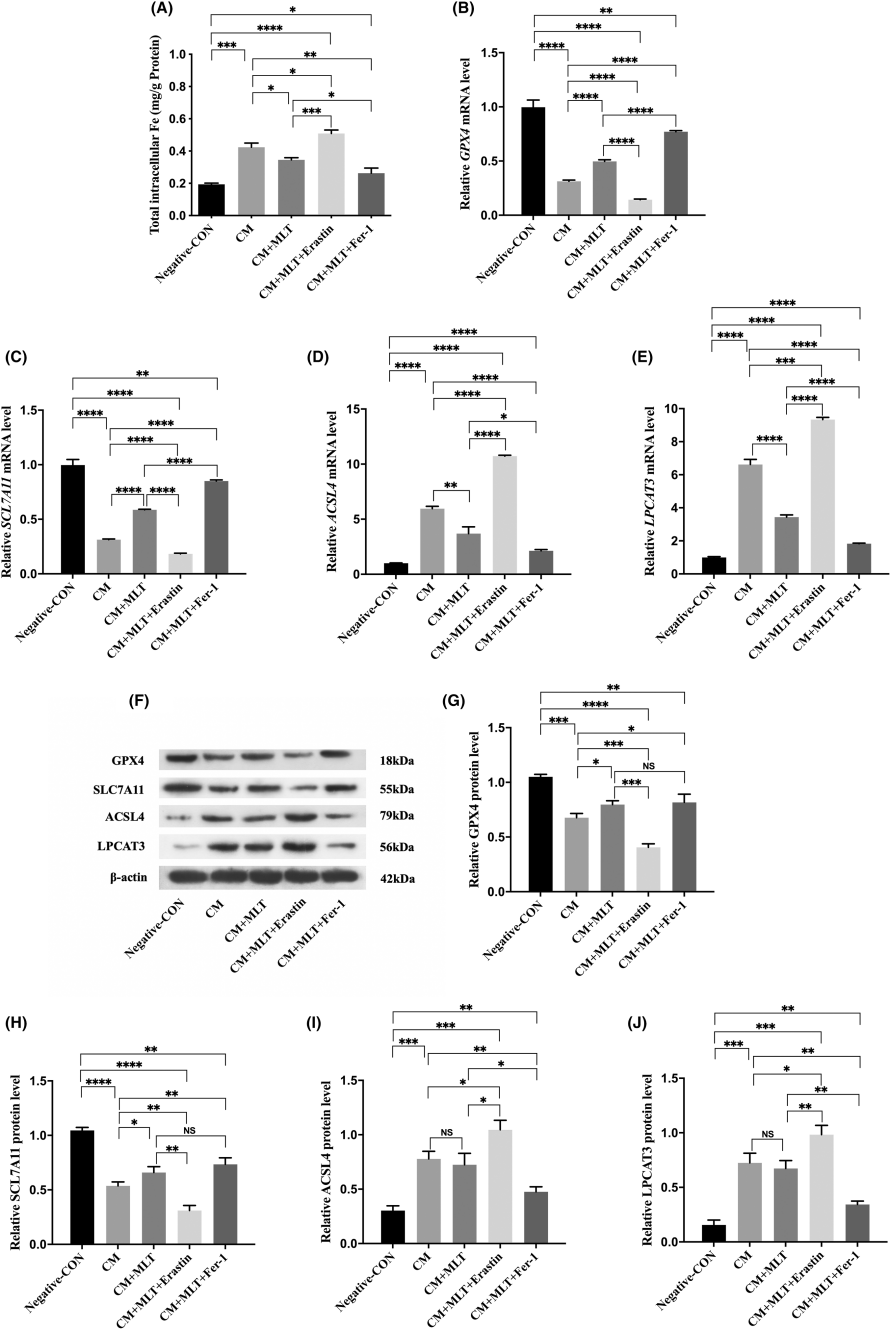
Ferroptosis occurred in CM‐induced nucleus pulposus (NP) cell injury and was regulated by melatonin (MLT), erastin, and Fer‐1. (A) The level of intracellular Fe was decreased after the treatment with MLT. The level of total intracellular Fe in NP cells was evaluated by the colorimetric Iron Assay kit (*n* = 3). (B–E) The relative mRNA levels of ferroptosis's markers in NP cells were measured by qPCR analyses (*n* = 3). (B) The level of GPX4 mRNA was increased after the treatment with MLT. (C) The level of SCL7A11 mRNA was improved after the treatment with MLT. (D) The level of ACSL4 mRNA was decreased after the treatment with MLT. (E) The level of LAPCAT3 mRNA was reduced after the treatment with MLT. (F–J) The relative protein levels of ferroptosis's markers in NP cells were measured by Western blot analyses (*n* = 3). (G) Quantitative analysis showed the increased level of GPX4 protein after the treatment with MLT. (H) Quantitative analysis showed the increased level of SCL7A11 protein after the treatment with MLT. (I and J) Quantitative analysis failed to show the changes of ACSL4 and LAPCAT3 protein levels after the treatment with MLT. Data are expressed as mean ± standard deviation. The two groups among the five groups are compared by using an independent *t*‐test. *p < 0.05, **p < 0.01, ***p < 0.001, ****p < 0.0001.

As the unique type of cell death, ferroptosis contributes to the pathogenesis of degenerative diseases.^35^ To investigate whether ferroptosis occurred in the CM‐induced NP cell injury, the ferroptosis‐associated markers were examined in the cell culture of NP cells after the treatment with CM from LPS‐stimulated RAW 264.7 macrophages for 24 h. Fe is a key regulator of ROS in the process of ferroptosis.^36^ The total intracellular Fe level in CM‐treated NP cells significantly increased compared with that in the untreated NP cells (Figure 4A). The expressions of the ferroptosis‐repressors^37^ GPX4 and SLC7A11 were significantly declined; however, the levels of the ferroptosis positively regulated enzymes like ACSL4 and LPCAT3 were increased in the CM‐treated NP cells when compared with those in untreated NP cells, as confirmed by the qPCR and Western blot (Figure 4B–J). Thus, the data indicated that ferroptosis was involved in the CM‐induced NP cell injury.

### 3.6 Suppression of ferroptosis contributes to the therapeutic potential of MLT

Next, to determine the protective effects of MLT against the CM‐induced ferroptosis in NP cells, NP cells were treated with CM in the presence or absence of 1 μM MLT. The results demonstrated that the level of total intracellular Fe was significantly decreased in the CM + MLT‐treated NP cells when compared with that in the CM‐treated NP cells (Figure 4A). The qPCR analysis revealed that mRNA levels of those ferroptosis‐associated markers including GPX4 and SLC7A11 were markedly upregulated, and ACSL4 and LPCAT3 were significantly downregulated in the CM + MLT‐treated NP cells when compared with those in CM‐treated NP cells (Figure 4B–E). These results were further confirmed by Western blot (Figure 4F–J). The results indicated that MLT could inhibit the ferroptosis of NP cells caused by the treatment with CM.

**FIGURE 5:**
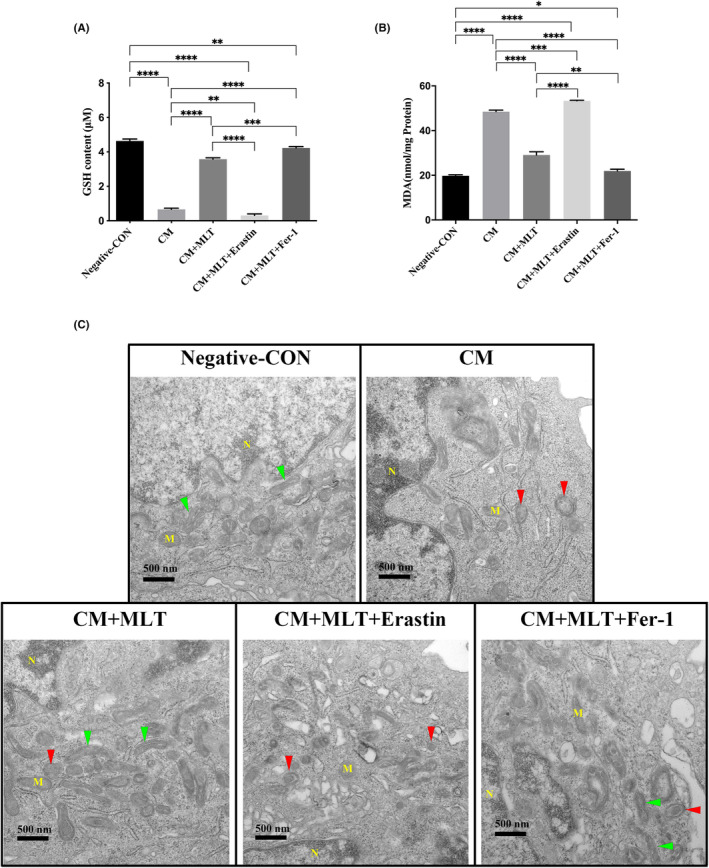
Inhibition of ferroptosis by the treatment with MLT. Nucleus pulposus (NP) cells were treated with conditioned medium (CM) in the presence or absence of MLT for 24 h, and followed by testing the effects of erastin and Fer‐1 on the levels of glutathione (GSH) and malondialdehyde (MDA). (A, B) The levels of GSH and MDA in NP cells were determined by ELISA (*n* = 3). (A) The level of GSH was markedly increased after the treatment with MLT, downregulated in combination with Erastin, and restored in the presence of Fer‐1. (B) The level of MDA was markedly decreased after the treatment with MLT, upregulated in combination with Erastin, and restored in the presence of Fer‐1. (C) TEM showed the morphological changes of mitochondria of NP cells. Data are expressed as mean ± standard deviation. The two groups among the five groups are compared by using an independent t‐test. The normal mitochondria were marked with green arrows, while the abnormal mitochondria with small sizes and thick membrane were marked with red arrows. *p < 0.05, **p < 0.01, ***p < 0.001, ****p < 0.0001.

To further investigate the association between MLT and ferroptosis, the ferroptosis inducer (erastin) and inhibitor (Fer‐1) were added to CM before the culture of NP cells. As shown in Figure 5 and Figure S1, GSH level significantly decreased (Figure 5A), and MDA (Figure 5B) and ROS (Figure S1) levels further increased in the CM + MLT + Erastin‐treated NP cells when compared with those in CM + MLT‐treated NP cells. While the reversal changes of GSH, as well as MDA and ROS levels, were achieved in CM + MLT + Fer‐1‐treated NP cells when compared with those in CM + MLT‐treated NP cells (Figure 5A,B and Figure S1). These results indicated that MLT could reduce NP cell injury and the inhibition effects of MLT were attenuated with erastin and enhanced with Fer‐1.

Compared with total intracellular Fe levels in the CM + MLT‐treated NP cells, the expressions of total intracellular Fe levels further decreased in the CM + MLT + Erastin‐treated NP cells, and the levels increased in the CM + MLT + Fer‐1‐treated NP cells (Fiuge 4A). Moreover, the markers of ferroptosis like GPX4 and SLC7A11 levels further decreased, and markers like ACSL4 and LPCAT3 levels further increased in the CM + MLT + Erastin‐treated NP cells when compared with those in the CM + MLT‐treated NP cells, in both the qPCR and Western blot analyses (Figure 4B–J). While these changes of GPX4 and SLC7A11 levels, as well as ACSL4 and LPCAT3 levels, were reversed in the CM + MLT + Fer‐1‐treated NP cells when compared with those in the CM + MLT‐treated NP cells (Figure 4B–J).

Due to mitochondria act as the major player during the ferroptosis,^35^ the morphological changes of mitochondria of NP cells were observed by using the electron microscopy. In comparison with the negative control group and the CM + MLT‐treated group, TEM results showed that the sizes of mitochondria in CM group were smaller, the mitochondrial membrane was thickened, and the numbers and length of mitochondrial crests were decreased and shorten respectively (Figure 5C, top right panel). In contrast, these morphological changes of mitochondria were markedly restored after the treatment with MLT (Figure 5C, bottom left panel), indicating that MLT attenuated the CM‐induced ferroptosis of NP cells. To further confirm this restorative effect of MLT on mitochondria, the ferroptosis inducer erastin and inhibitor Fer‐1 were administrated in combination with MLT treatment respectively. In comparison with CM + MLT‐treated group, the morphological changes of mitochondria were aggravated in the CM + MLT + Erastin treated group (Figure 5C, bottom middle panel). However, mitochondrial morphologies were normalized in the CM + MLT + Fer‐1‐treated group (Figure 5C, bottom right panel and Figure S1). Taken together, the data indicated that MLT could protect NP cells against the CM‐induced inflammation through the inhibition of ferroptosis.

## 4 DISCUSSION

IDD is considered a complex multifactorial process involving multiple pathways that are influenced by both genetic and environmental factors. Macrophage infiltration plays an important role in the occurrence and progression of IDD.^38^, ^39^, ^40^, ^41^ Macrophages were found to readily appear in the degenerated IVD instead of the healthy IVD,^38^, ^39^, ^40^, ^41^ and the number of inflammatory type 1 macrophages (M1) correlated with IDD progression.^38^, ^39^ Hwang et al. found that human NP cells exposed to a macrophage‐CM showed a dramatic increase in the expressions of inflammatory cytokines (IL‐6 and IL‐8) and key matrix catabolic molecules (MMP‐1 and MMP‐3) compared with naïve NP cells.^42^ Yang et al. found that rat NP cells exposed to conditioned media from RAW 264.7 macrophages with IFN‐γ stimulation dramatically increased expression levels of inflammatory cytokines (IL‐1β, TNF‐α, COX‐2) and key matrix catabolic molecules (MMP‐3, MMP‐13, ADAMTS‐4, ADAMTS‐5).^7^ In line with these reports, current studies demonstrated that the CM from LPS‐stimulated RAW 264.7 macrophages could initiate molecular changes associated with IDD, including upregulated expression of inflammation‐related genes (IL‐6, IL‐1β, TNF‐α, COX‐2, iNOS), increased intracellular oxidative stress (increased ROS and MDA levels, but decreased GSH levels), and disrupted the homeostasis of ECM increased expression of key matrix catabolic molecules (MMP‐13, ADAMTS‐4, ADAMTS‐5), reduced the expression of major matrix anabolic molecules (ACAN and COL2A1) in NP cells. That is to say, secreted factors from LPS‐stimulated RAW 264.7 macrophages could initiate and aggravate NP cell injury and IDD. Therefore, results from the current study shed light on targeting suppression of macrophage infiltration for preventing NP cell injury and ameliorating IDD‐related lower back pain.

In the present study, we found that MLT could alleviate the CM‐induced NP cell injury in a dose‐dependent manner. MLT is an indoleamine hormone involved in various diseases, including lung injury,^43^ renal fibrosis,^44^ diabetes,^20^ nonalcoholic fatty liver disease,^14^ Alzheimer's disease,^45^ Parkinson disease^46^ and cancer.^47^ The role of MLT in IDD was first discovered by Turgut et al. that surgical removal of the pineal gland in chickens reduced their serum MLT levels and accelerated the deterioration of IDD.^30^ Since then, an increasing number of studies have begun to focus on the role of MLT on IDD. Elfering et al. have shown that night shift workers are more likely to suffer from IDD.^48^ Notably, Tian et al. studied in human patients with IDD found that plasma MLT concentrations were significantly decreased and associated with disease duration, disease severity, and inflammatory cytokines levels.^24^ The relationship between MLT and IDD was further supported by in vivo MLT administration experiments, which showed that exogenous MLT administration could activate the recovery process in the degenerated IVD tissue in rats^3^ and rabbits.^29^ Moreover, in vitro *s*tudies performed by Li et al. have confirmed the existence of MLT membrane receptors (MT1 and MT2) in IVD tissues and NP cells, and found that MLT acted on MT1/2 and subsequently inhibited NP cell proliferation and ECM remodelling in a dose‐dependent manner.^49^ Infiltrating macrophages play important roles in the initiation and exacerbation of IDD.^38^, ^39^, ^40^, ^41^ Previous studies showed that MLT could combine with its nuclear receptor (retinoid receptor‐related orphan receptor, ROR), thus inducing the phenotypic changes of inflammatory M1 to anti‐inflammatory type 2 macrophages (M2)^50^ and inhibiting inflammatory‐related gene expressions.^51^ In our current study, MLT could alleviate CM‐induced NP cell injury in a dose‐dependent manner, including decreased intracellular oxidative stress (decreased ROS and MDA levels, but increased GSH levels), downregulated expression of inflammation‐related factors (IL‐6, IL‐1β, TNF‐α, COX‐2, iNOS), decreased expression of key matrix catabolic molecules (MMP‐13), induced the expression of major matrix anabolic molecules (ACAN, COL2A1) in NP cells. All of these findings, together with our data demonstrated that MLT may serve as a promising therapeutic strategy for IDD.

Despite accumulating evidence demonstrated the benefits of MLT in IDD, considerable work still needs to be done on the understanding the mechanisms before the application of MLT as the clinical treatment for IDD. A multitude of mechanisms has been forwarded to exert the beneficial effects of MLT in IDD, including anti‐oxidant, anti‐inflammatory, anti‐apoptosis, promote autophagy in IVD tissues and NP cells.^23^ Recently, MLT has been identified as an effective inhibitor of ferroptosis and its anti‐ferroptosis provided a potential therapeutic target for treating acute sleep deprivation‐induced cognitive impairment,^31^ traumatic brain injury,^32^ as well as PM 2.5‐induced lung injury.^43^ To our knowledge, this is the first time to explore the anti‐ferroptosis effect of MLT in NP cells. Ferroptosis, discovered by Stockwell et al. in 2012, is an iron‐dependent regulated cell death, which is morphologically, biochemically and genetically distinct from apoptosis, autophagy, and necrosis.^8^ Iron, as a constituent of iron–sulphur proteins, hemoproteins, myoglobin and cytochrome p450 system, is essential for the accumulation of lipid peroxides in the development of ferroptosis.^52^ The current study found that compared with negative control cells, the intracellular iron levels and ferroptosis's marker proteins GPX4, SLC7A11, GCLC and NRF2 expression levels significantly declined, ACSL4, LPCAT3 increased, and the microstructure of mitochondria changed in the CM‐treated NP cells, which indicated the involvement of ferroptosis in IDD. In support of our result, Yang et al. studied in human IVD tissues found that the ferroptosis's marker proteins GPX4 and FTH expression levels significantly decreased, while PTGS2 was increased in the degenerated IVD tissues compared with the normal tissues.^11^ In vivo studies showed that treatment with iron chelator DFO or ferroptosis inhibitors Fer‐1 ameliorated IDD progression compared to the saline group.^10^, ^11^ In our study, MLT ameliorated NP cell injury and ferroptosis was involved in this process. Well the detail mechanism and pathways should further be investigated in future studies.

There were several limitations in this study. First, although multiple ferroptosis‐related molecules such as GPX4, SLC7A11, ACSL4 and LPCAT3 were involved in reducing NP cell injury using MLT, the exact and detail mechanisms and pathways of ferroptosis during this process should further be explored in future studies. Second, the cell experiments in this study were performed to explore the relationship between MLT, IDD and ferroptosis, however, animal experiments should be conducted in future. In addition, this study lacked the exploration about the influence of higher concentration of MLT. Subsequent studies on this part may be helpful to understand the effects of higher concentration of MLT in IDD. Finally, in this study, we focused on the effects of MLT treatment on NP cells other than macrophages, therefore our study did not investigate the exact components in CM from LPS‐stimulated RAW264.7 macrophages, which should also be further studied in future.

In summary, current studies demonstrated that MLT treatment could markedly protect NP cells against the inflammatory injuries caused by the CM from the LPS‐stimulated RAW264.7 macrophages, partially through the inhibition of ferroptosis of NP cells. This finding provides substantial evidence revealing the ferroptosis involved in the pathogenesis of IDD, and highlights MLT treatment as a potential therapeutic approach for the clinical management of IDD.

## AUTHOR CONTRIBUTIONS

**Xinyu Dou:** Conceptualization (equal); data curation (equal); methodology (equal); writing – original draft (equal); writing – review and editing (equal). **Yunlong Ma:** Conceptualization (equal); methodology (equal); validation (equal); writing – review and editing (equal). **Qipeng Luo:** Conceptualization (equal); data curation (equal); validation (equal); writing – original draft (equal). **Chunyu Song:** Data curation (equal). **Mei‐Juan Liu:** Data curation (equal). **Xiao Liu:** Data curation (equal). **Donglin Jia:** Formal analysis (lead); supervision (equal). **Shuiqing Li:** Conceptualization (equal); supervision (equal). **Xiaoguang Liu:** Conceptualization (equal); project administration (lead); writing – review and editing (equal).

## FUNDING INFORMATION

This research was funded by the Beijing Municipal Science and Technology Commission, Administrative Commission of Zhongguancun Science Park to XG.L.(grant number: Z191100007619023) and the National Natural Science Foundation of China to XG.L. (grant number: 81972103).

## CONFLICT OF INTEREST STATEMENT

The authors confirm that there are no conflicts of interest.

## Supporting information


Figure S1:

## Data Availability

The data that support the findings of this study are available from the corresponding author upon reasonable request.
