# Supplementary figures and images for: Therapeutic potential of melatonin in the intervertebral disc degeneration through inhibiting the ferroptosis of nucleus pulpous cells
Source: J Cell Mol Med. 2023 Jun 16;27(16):2340–53. doi: 10.1111/jcmm.17818 (PMC10424295; doi:10.1111/jcmm.17818)

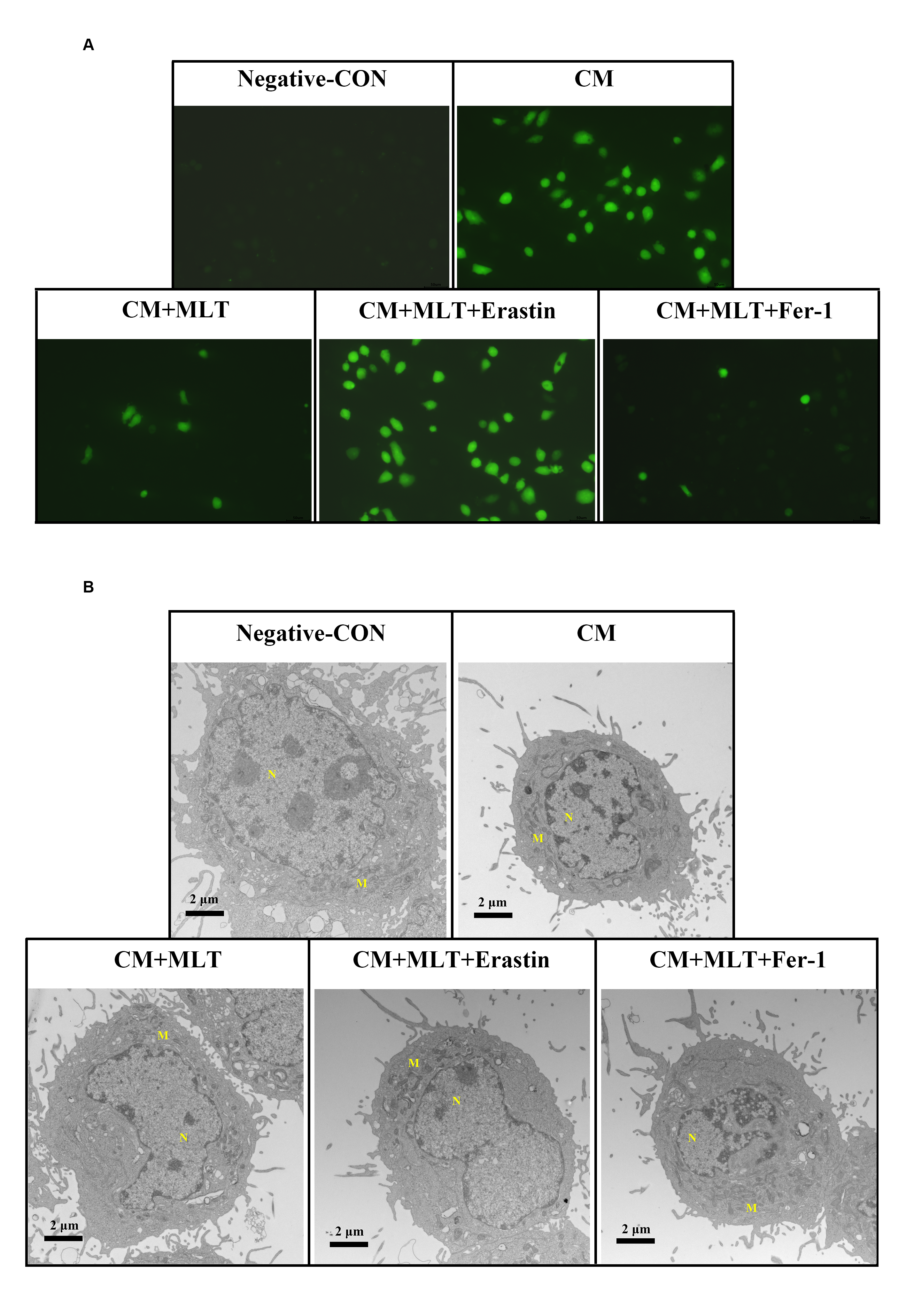

Supplement: Supplementary file 1 — Figure S1: [file JCMM-27-2340-s001.tif]
